# Assessment of adherence to public health measures and their impact on the COVID-19 outbreak in Benin Republic, West Africa

**DOI:** 10.11604/pamj.2021.38.293.26843

**Published:** 2021-03-22

**Authors:** Philippe Sessou, Joseph Nelson Siewe Fodjo, Charles Sossa Jérôme, Souaïbou Farougou, Robert Colebunders

**Affiliations:** 1 Research Unit of Communicable Diseases, Polytechnic School of Abomey-Calavi, University of Abomey-Calavi, 01 BP 2009, Cotonou, Benin Republic,; 2 Global Health Institute, University of Antwerp, Doornstraat 331, 2610 Antwerp, Belgium,; 3 Division of Health Promotion, Regional Institute of Public Health, Ouidah, Benin

**Keywords:** COVID-19, preventive measures, adherence, Benin, epidemiology

## Abstract

**Introduction:**

following the global COVID-19 outbreak, the government of Benin implemented preventive measures to stall viral transmission. We sought to evaluate adherence of the Beninese people to these preventive measures, in order to identify predictors of poor adherence and adapt the national response to COVID-19.

**Methods:**

two consecutive online surveys were conducted between May and August 2020. Four hundred and sixty two and 507 adult participants aged 18 years and above responded to the first and second survey respectively, with >70% being males

**Results:**

more than 98% of respondents reported wearing face masks. A five-point adherence score was constituted by scoring observance to key preventive measures (mask use, physical distancing, hand hygiene, coughing hygiene and avoiding to touch one´s face). We observed that the mean adherence scores were fairly stable over time, respectively 4.08 and 4.03 during the first and second survey (p=0.439). Increasing age (aOR=1.043, 95% CI: 1.026 - 1.061; p<0.001) and obtaining COVID-19 information from official sources (aOR=1.628, 95% CI: 1.275 - 2.081; p<0.001) were significantly associated with higher adherence scores in a multivariable model.

**Conclusion:**

these findings suggest that a wide dissemination of adequate information about COVID-19 would increase adherence, and that targeted efforts should be directed towards increasing the compliance to preventive measures among the younger age groups

## Introduction

Since its initial outbreak in China in December 2019, the novel coronavirus disease 2019 (COVID-19) has spread across nations and continents affecting over 42 million people and causing more than a million deaths. The West African nation of Benin has not been spared by this pandemic; in fact, as of the 25 October 2020, 2,643 confirmed cases of COVID-19 had occurred in Benin, with 41 deaths [1]. Unlike most nations which implemented stringent confinement measures to contain the outbreak, the Benin government wanted to avoid the huge socio-economic impacts of a national lockdown since the majority of its population is involved in the informal sector. Therefore, they opted for a sanitary cordon around the affected regions with free movement of people while respecting hand hygiene and barrier measures such as the wearing of masks and the respect of physical distancing [2]. These measures were officially instituted on March 16, 2020, when the country already counted one case of the disease. Mitigation measures also consisted of the closure of churches, mosques, clubs, and bars while restaurants and supermarkets were left open. Earlier during the pandemic (February 2020), the Ministry of Health had installed a temperature scanner, handwashing apparatus, and an isolation room in the country´s international airport distribution to screen all flight passengers coming into Benin, since air traffic is a major route in the spread of COVID-19 [3].

As part of the easing of COVID-19 response measures, the Benin Government decided after consultation with religious leaders to authorize the reopening of worship places (churches, mosques, temples, etc.) throughout the national territory from June 2, 2020 and this in strict compliance with official instructions. In worship places such as convents and temples of endogenous religions, religious authorities such as priests, dignitaries and followers were invited to scrupulously ensure the application of barrier measures and strict hygiene. Hugs, handshakes, and other forms of contact were also to be avoided. Furthermore, religious authorities were instructed to ensure that reasonable time margins are observed between celebrations and that worship places are regularly disinfected. Bars were also allowed to reopen at that same date. To this end, their promoters and managers were required to comply with the following preventive measures: the installation of hand washing or sanitizing facilities, and the observance of a safe distance of at least one meter between users. However, discotheques and festive ceremonies were forbidden until further notice. Beaches were also to remain closed to the public [4]. With the varying information about COVID-19 circulating on different platforms (TV, radio, social media, friends and relatives), people are likely to have diverging opinions about the disease and the mitigation strategies deployed by the government. It is expected that different perceptions of COVID-19 and associated preventive measures would influence the level of adherence to the latter. Therefore, it is important to assess the adherence of the Beninese population to the preventive strategies issued by the government, as well as their effectiveness in preventing COVID-19 transmission throughout the country. The purpose of this study was to investigate how well adult residents of Benin have taken up these health measures that are crucial in preventing the spread of the virus.

## Methods

**Study setting and procedures:** the study was carried out in Benin Republic, West Africa. Two consecutive cross-sectional surveys investigating the adherence to COVID-19 preventive measures were conducted. The first took place from May 25 to June 17, 2020, whereas the second was held from July 23 to August 22, 2020. Data were collected through an online survey initiated by the International Citizen project on COVID-19 (ICPCOVID) consortium. A secure website was used to design and host a questionnaire, which was developed to investigate individual/community factors that may influence adherence to COVID-19 preventive measures. The website interface was designed to be easily accessible by various devices such as computers, tablets, and smart phones. Eligible participants were Beninese aged 18 years or older, who were able to read and understand French, and residing in Benin at the time of data collection. The web link to the online survey was shared via various social media platforms to relatives, friends, and colleagues; consenting volunteers submitted their responses anonymously. The information collected included sociodemographic characteristics, health status and determinants of health, adherence to individual or community preventive measures, consequences of the COVID-19 pandemic on people´s lives. In addition, the questionnaire also included questions about the presence or absence of flu-like symptoms experienced by the respondents during the past fourteen days (prior to responding to the questionnaire). Participants were asked whether they had been tested for COVID-19, and for the results of the test if available. Submitted responses were stored in the secure ICPCOVID website until data extraction.

**Ethical considerations:** the study protocol was approved by the Ethical committee of the University of Antwerp, Belgium under N°20/13/148 of 23 March 2020 and by the National Ethics Committee for Health Research (CNERS) of Benin (ethical opinion N°21 of 7 May 2020). Anonymity was ensured and informed e-consent was required from each participant before submitting responses, which were securely stored in a password-protected server in Belgium.

**Data processing and analysis:** data analysis was performed using the software R, version 3.6.2. A 5-point adherence score was constructed based on the respondents´ adherence to the following preventive measures: mask use, physical distancing, hand hygiene (regular hand washing or use of alcohol-based hand gel), coughing hygiene, and avoiding to touch one´s face. Observance of each measure was scored as 1 point, and otherwise zero; the final score ranged from 0-5 with a higher score implying higher adherence to the preventive measures. Descriptive statistics were presented using means with standard deviation (SD) for continuous outcomes, and percentages (%) for categorical variables. Pearson´s Chi-squared test was used to investigate associations between categorical variables, and to compare proportions across the surveys. Continuous variables were assessed for normality using the Shapiro-Wilk test, and compared across groups using parametric tests (t-test, ANOVA) or non-parametric tests (Mann-Whitney U, Kruskal-Wallis) as appropriate. For multivariable analysis, we constructed an ordinal logistic regression model to investigate factors associated with adherence to national preventive measures against COVID-19, using the adherence score as the dependent variable. P-values < 0.05 were considered statistically significant.

## Results

**Participants´ characteristics during the two surveys in Benin:** after data cleaning and application of inclusion criteria, 462 and 507 responses were kept respectively for the first round and the second round surveys. The characteristics of participants are summarized in Table 1. The study population with average ages 28.9 (9.05) in the first round and 30.9 (8.98) in the second round were predominantly male (73-75%) and of the Christian religion (77.3-79%). Furthermore, almost all respondents had reached a university level of education. The proportion of people tested for COVID-19 in the second round (18.7%) was higher than in the first round (4.1%); p<0.001. However the prevalence of COVID-19 among tested individuals was not significantly different in both surveys (5.56% in survey 1 and 3.41% in survey 2; p=0.531).

**Table 1:** participants´ characteristics during the two surveys in Benin Republic

Characteristics	Survey 1	Survey 2	p-value
	N=462	N=507	
**Age: mean (SD)**	28.9 (9.05)	30.9 (8.98)	<0.001
**Gender: n (%)**			0.495
Female	115 (24.9%)	137 (27.0%)	
Male	347 (75.1%)	370 (73.0%)	
**Education: n (%)**			<0.001
Primary	0 (0.00%)	8 (1.58%)	
Secondary	48 (10.4%)	50 (9.86%)	
Undergraduate	228 (49.4%)	184 (36.3%)	
Postgraduate	186 (40.3%)	265 (52.3%)	
**Religion: n (%)**			<0.001
Christian	357 (77.3%)	403 (79.5%)	
Muslim	87 (18.8%)	57 (11.2%)	
None	5 (1.08%)	19 (3.75%)	
Other	13 (2.81%)	28 (5.52%)	
**Marital status: n (%)**			0.182
Single	301 (65.2%)	296 (58.4%)	
Cohabitation	51 (11.0%)	69 (13.6%)	
Married	107 (23.2%)	137 (27.0%)	
Widow	3 (0.65%)	5 (0.99%)	
**Residence: n (%)**			0.232
Rural	55 (11.9%)	64 (12.6%)	
Sub-urban	196 (42.4%)	188 (37.1%)	
Urban	211 (45.7%)	255 (50.3%)	
**Profession: n (%)**			<0.001
Jobless	38 (8.23%)	55 (10.8%)	
Private	64 (13.9%)	106 (20.9%)	
Public	103 (22.3%)	117 (23.1%)	
Self-employed	38 (8.23%)	56 (11.0%)	
Student	219 (47.4%)	173 (34.1%)	
**Health sector: n (%)**			0.012
No	292 (63.2%)	360 (71.0%)	
Yes	170 (36.8%)	147 (29.0%)	
**Flu symptoms: n (%)**			0.075
No	284 (61.5%)	282 (55.6%)	
Yes	178 (38.5%)	225 (44.4%)	
**Chronic disease n (%)**			0.052
No	417 (90.3%)	436 (86.0%)	
Yes	45 (9.74%)	71 (14.0%)	
**Tested for COVID-19: n (%)**			<0.001
No	443 (95.9%)	412 (81.3%)	
Yes	19 (4.11%)	95 (18.7%)	
**COVID-19 test result: n (%)**			0.531
Negative	17 (94.4%)	85 (96.6%)	
Positive	1 (5.56%)	3 (3.41%)	
Not available	1	7	

**Adherence to personal preventive measures in Benin during both surveys:** considering the individual COVID-19 preventive measures, the mean adherence scores slightly decreased between the two surveys (from 4.08 to 4.03), albeit being non-significant (Table 2). Wearing mask had the highest adherence rate (98.0-98.8%). Adherence to physical distancing, regular hand washing using soap and water, covering mouth when coughing, and using gel were also high with rates reaching 72.7-75.1% for physical distancing, 87-92% for regular washing hand and 53.2-57.0% for using hand gel. However, adherence regarding temperature measurement was low (19.0 and 21.1%) during both surveys. The proportion of people who believed that lockdown is needed in Benin to contain the pandemic in the second round (51.9%) was significantly lower compared to the first round (68.2%); p<0.001. With regard to travelling, the proportion of individuals who reported travelling during the 7 days preceding their participation to the study significantly increased between the first and second surveys (p<0.001). Participants were not worried much about their health and for their loved ones during the pandemic, with mean Likert scores for worriedness ranging between 1 - 2 on a scale of 1 to 5.

**Table 2:** personal preventive behaviors in Benin during surveys 1 and 2

Characteristics	Survey 1	Survey 2	p-value
	N=462	N=507	
**Adherence score (Likert: 0-5): mean (SD)**	4.08 (1.05)	4.03 (1.08)	0.439
**Mask use: n (%)**			0.484
No	9 (1.96%)	6 (1.19%)	
Yes	451 (98.0%)	498 (98.8%)	
**Physical distancing: n (%)**			0.433
No	126 (27.3%)	126 (24.9%)	
Yes	336 (72.7%)	381 (75.1%)	
**Cover mouth when coughing: n (%)**			0.586
No	60 (13.0%)	73 (14.4%)	
Yes	402 (87.0%)	434 (85.6%)	
**Wash hands after coughing n (%)**			0.689
No	190 (41.1%)	216 (42.6%)	
Yes	272 (58.9%)	291 (57.4%)	
**Regular temperature measurement: n (%)**			0.473
No	374 (81.0%)	400 (78.9%)	
Yes	88 (19.0%)	107 (21.1%)	
**Wash hands regularly: n (%)**			0.015
No	37 (8.01%)	66 (13.0%)	
Yes	425 (92.0%)	441 (87.0%)	
**Use hand gel: n (%)**			0.267
No	216 (46.8%)	218 (43.0%)	
Yes	246 (53.2%)	289 (57.0%)	
**Avoid touching face: n (%)**			0.282
No	200 (43.3%)	238 (46.9%)	
Yes	262 (56.7%)	269 (53.1%)	
**Disinfect phone upon returning home: n (%)**			0.206
No	339 (75.0%)	391 (78.7%)	
Yes	113 (25.0%)	106 (21.3%)	
**Stay home if flu symptoms: n (%)**			0.455
No	74 (37.8%)	112 (41.6%)	
Yes	122 (62.2%)	157 (58.4%)	
**Meeting with ≥50 persons: n (%)**			<0.001
No	417 (90.3%)	392 (77.3%)	
Yes	45 (9.74%)	115 (22.7%)	
**Difficulty to remain at home (Likert: 1-5): Mean (SD)**	3.31 (1.33)	3.41 (1.40)	0.223
**Last physical contact with people outside household: n (%)**			0.017
Today	115 (24.9%)	91 (17.9%)	
2 days ago	51 (11.0%)	71 (14.0%)	
3-6 days	34 (7.36%)	59 (11.6%)	
≥7days	89 (19.3%)	91 (17.9%)	
No contact	173 (37.4%)	195 (38.5%)	
**Worry about one's own health (Likert: 1-5): Mean (SD)**	2.06 (1.33)	1.99 (1.26)	0.372
**Worry about others´ health (Likert: 1-5): Mean (SD)**	2.10 (1.36)	1.99 (1.26)	0.170
**Lockdown needed in Benin? n (%)**			<0.001
No	147 (31.8%)	244 (48.1%)	
Yes	315 (68.2%)	263 (51.9%)	

**Details regarding face mask use:** while most respondents reported using masks all the time when they left their residence (74.9% in Survey 1 and 79.5% in survey 2), a minority also wore masks at home (7.54% in Survey 1 and 8.03% in Survey 2). The main type of mask used by the Beninese during the pandemic were reusable cloth (fabric) masks (Table 3). For the 15 respondents who reported not using face masks during the surveys, the reasons given included the fact that masks made them uncomfortable (46.7%), lack of financial means to acquire masks in 13.3%, while another 40.0% of them felt that wearing a mask was not necessary during the COVID-19 outbreak.

**Table 3:** details about mask for COVID-19 prevention in Benin

Characteristics: n (%)		Survey 1	Survey 2	p-value
		N=451	N=498	
**Mask type**				0.013
	Cloth	266 (59.0%)	315 (63.3%)	
	Disposable	185 (41.0%)	177 (35.5%)	
	Professional	0 (0.00%)	6 (1.20%)	
**Wear mask in crowds**				0.892
	No	84 (18.6%)	90 (18.1%)	
	Yes	367 (81.4%)	408 (81.9%)	
**Wear mask in public transport**				0.012
	No	183 (40.6%)	162 (32.5%)	
	Yes	268 (59.4%)	336 (67.5%)	
**Wear mask at work**				0.022
	No	242 (53.7%)	229 (46.0%)	
	Yes	209 (46.3%)	269 (54.0%)	
**Wear mask sometimes in public**				0.841
	No	214 (47.5%)	232 (46.6%)	
	Yes	237 (52.5%)	266 (53.4%)	
**Wear mask all the times in public**				0.109
	No	113 (25.1%)	102 (20.5%)	
	Yes	338 (74.9%)	396 (79.5%)	
**Wear mask at home**				0.871
	No	417 (92.5%)	458 (92.0%)	
	Yes	34 (7.54%)	40 (8.03%)	

**Adherence to community preventive measures in response to COVID-19 threat:** during the surveys, the majority of participants responded that they had not travelled, avoided going to a religious gathering, a public gym, a restaurant, a club, family gathering with high adherence rates varying between 88.8% and 99.8% (Table 4). The proportions of participants who went to parties, religious gatherings, and who were in a vehicle with more than three other persons were significantly higher in the second survey compared to the first. Moreover, half of the participants had visited a market in the past seven days and nearly half continued to visit beauty salon. Globally, adherence to community preventive measures decreased with time.

**Table 4:** adherence to community preventive measures against COVID-19

Characteristics (for the past 7 days): n (%)	Survey 1	Survey 2	p-value
	N=462	N=507	
**Visited restaurant**			0.390
No	454 (98.3%)	493 (97.2%)	
Yes	8 (1.73%)	14 (2.76%)	
**Visited bar**			0.925
No	456 (98.7%)	499 (98.4%)	
Yes	6 (1.30%)	8 (1.58%)	
**Visited club**			0.608
No	460 (99.6%)	506 (99.8%)	
Yes	2 (0.43%)	1 (0.20%)	
**Attended party**			<0.001
No	458 (99.1%)	481 (94.9%)	
Yes	4 (0.87%)	26 (5.13%)	
**Attended funeral**			0.031
No	454 (98.3%)	485 (95.7%)	
Yes	8 (1.73%)	22 (4.34%)	
**Attended religious gathering**			<0.001
No	456 (98.7%)	450 (88.8%)	
Yes	6 (1.30%)	57 (11.2%)	
**Attended family gathering**			0.015
No	459 (99.4%)	492 (97.0%)	
Yes	3 (0.65%)	15 (2.96%)	
**Attended sport event**			0.969
No	454 (98.3%)	497 (98.0%)	
Yes	8 (1.73%)	10 (1.97%)	
**Visited gym**			0.061
No	448 (97.0%)	478 (94.3%)	
Yes	14 (3.03%)	29 (5.72%)	
**Visited beauty salon**			0.996
No	258 (55.8%)	282 (55.6%)	
Yes	204 (44.2%)	225 (44.4%)	
**Went to market**			0.924
No	230 (49.8%)	255 (50.3%)	
Yes	232 (50.2%)	252 (49.7%)	
**Was in car with 3 other persons**			0.001
No	321 (69.5%)	301 (59.4%)	
Yes	141 (30.5%)	206 (40.6%)	
**Travelled**			0.001
No	339 (73.4%)	321 (63.3%)	
Yes	123 (26.6%)	186 (36.7%)	

**Flu-like symptoms reported by participants in both surveys:** globally, respectively 25.3% and 28.0% participants in the first and second surveys reported that they were experiencing at least one flu-like symptom during the past 14 days. Headache was the most frequent symptom in both surveys followed by fever, weakness and coryza (Table 5). The proportions of participants with fever and shortness of breath were significantly higher in survey 2 (respectively 14.0% and 3.35%) than in survey 1 (respectively 8.8% and 1.08%); p<0.05

**Table 5:** flu like symptoms reported by participants in both surveys

Characteristics n (%)	Survey 1	Survey 2	p-value
	N=462	N=507	
**Fever**			0.017
No	421 (91.1%)	436 (86.0%)	
Yes	41 (8.87%)	71 (14.0%)	
**Loss of smell**			0.167
No	458 (99.1%)	496 (97.8%)	
Yes	4 (0.87%)	11 (2.17%)	
Dry cough			0.847
No	453 (98.1%)	499 (98.4%)	
Yes	9 (1.95%)	8 (1.58%)	
**Productive cough**			0.177
No	442 (95.7%)	474 (93.5%)	
Yes	20 (4.33%)	33 (6.51%)	
**Shortness of breath**			0.031
No	457 (98.9%)	490 (96.6%)	
Yes	5 (1.08%)	17 (3.35%)	
**Sore throat**			0.921
No	439 (95.0%)	480 (94.7%)	
Yes	23 (4.98%)	27 (5.33%)	
**Coryza**			0.381
No	424 (91.8%)	456 (89.9%)	
Yes	38 (8.23%)	51 (10.1%)	
**Headaches**			0.384
No	345 (74.7%)	365 (72.0%)	
Yes	117 (25.3%)	142 (28.0%)	
**Weakness**			0.153
No	409 (88.5%)	432 (85.2%)	
Yes	53 (11.5%)	75 (14.8%)	
Loss of taste:			0.767
No	455 (98.5%)	497 (98.0%)	
Yes	7 (1.52%)	10 (1.97%)	
**Body pains**			0.168
No	436 (94.4%)	466 (91.9%)	
Yes	26 (5.63%)	41 (8.09%)	
Nausea:			0.235
No	456 (98.7%)	494 (97.4%)	
Yes	6 (1.30%)	13 (2.56%)	
Diarrhea:			0.934
No	447 (96.8%)	489 (96.4%)	
Yes	15 (3.25%)	18 (3.55%)	
**Chest pain**			0.838
No	445 (96.3%)	486 (95.9%)	
Yes	17 (3.68%)	21 (4.14%)	
**Loss of appetite**			1.000
No	452 (97.8%)	495 (97.6%)	
Yes	10 (2.16%)	12 (2.37%)	

**Factors associated with adherence to COVID-19 preventive measures:** a multivariable model investigating factors associated with the composite adherence score revealed that obtaining COVID-19 information from official sources (government websites, radio, television) significantly increased the odds for a higher adherence score (aOR = 1.675, 95% CI: 1.295 - 2.170; p<0.001), same as increasing age (aOR = 1.043, 95% CI: 1.026 - 1.061; p<0.001); Table 6. Furthermore, having experienced flu-like symptoms in the past 14 days, and residing in sub-urban/urban settings were associated with lower adherence scores

**Table 6:** ordinal logistic regression on adherence score

Covariate	OR	2.5 %	97.5 %	p value
Age	1.0431328	1.0257266	1.0612718	<0.001
Male gender	0.9315381	0.7057275	1.2272822	0.615
Education: primary	Reference			
Education: secondary	1.6993601	0.3405512	7.9815777	0.503
Education: undergraduate	1.5198216	0.3156742	6.8649170	0.587
Education: postgraduate	1.3613463	0.2837213	6.1170075	0.688
Residence: rural	Reference			
Residence: sub-urban	0.5932307	0.3916522	0.8929559	0.013
Residence: urban	0.6450014	0.4284817	0.9648486	0.034
Worry about my health (Likert score)	0.9649133	0.8727902	1.0668776	0.485
Worry about others' health (Likert score)	1.1297148	1.0209753	1.2513722	0.019
Socioeconomic category: low	Reference			
Socioeconomic category: lower middle	0.7984449	0.5593032	1.1359727	0.212
Socioeconomic category: upper middle	1.2300741	0.7829310	1.9333601	0.369
Socioeconomic category: high	0.6298129	0.2914557	1.3826756	0.243
Flu-like symptom(s) during past 14 days	0.5102300	0.3987981	0.6520044	<0.001
Health sector student/worker	0.9085262	0.7007665	1.1789918	0.470
Difficulty to stay home (Likert score)	0.9734692	0.8897259	1.0648076	0.557
COVID-19 information from social media	0.9705976	0.6389187	1.4664629	0.888
COVID-19 information from official source	1.6284735	1.2751208	2.0811522	<0.001
Survey round 1	Reference			
Survey round 2	0.9234922	0.7240331	1.1774973	0.521

## Discussion

To reduce the COVID-19 transmission and its impacts, high adherence to public health measures is crucial [5]. The present study has accessed the adherence to public health measures by the population of Benin to mitigate COVID-19 transmission via two consecutive online surveys. The study has revealed that obtaining COVID-19 information from official government sources was significantly associated with increased adherence of the population. Also, the better adherence to COVID-19 preventive measures among older respondents is most likely explained by the fact that older people were aware of their increased risk for severe COVID-19 disease [6]. The fact that adherence increased with age highlights the need for targeted interventions to improve preventive behavior among the younger age groups as they can become infected and transmit the disease to the high-risk groups (elderly and chronically sick individuals) even if themselves are at low risk [7]. Over time, adherence to individual preventive measures against COVID-19 seemed fairly stable, but community measures (such as going to bars, restaurants, travelling, etc.) were decreasingly respected. This could be because prior to the second survey, the Benin Government authorized the reopening of worship places (churches, mosques, temples, etc.), bars/restaurants, opening of the markets and allowing people to travel as part of easing the COVID-19 response. However, the fact that people continued to adhere to personal preventive measures suggests that they were aware of the need to remain careful despite the lifting of the restrictions for socio-economic reasons. About 98% of the respondents reported wearing face masks when going out. This proportion is very high compared to what was reported during similar online surveys in other low-to-middle-income-countries (LMIC) such as Brazil (45.7%), in Democratic Republic of Congo (43.2%) Somalia (56.2%) and Uganda (32.7%) [8,9]. Mathematical models have suggested using surgical masks with a satisfactory population coverage (>80%) as reported in our survey would result in a rapid control of COVID-19 transmission [10]. Whether high mask use has indeed played a role in the reported low disease burden and mortality due to COVID-19 in Benin needs further investigation.

It is important to note that during the second survey a higher percentage of responders reported fever with shortness of breath. This may be caused by the lifting of the COVID-19 preventive measures by the government and an increase in COVID-19 infections via community transmission. Also, a higher percentage of respondents of survey 2 reported to have been tested for COVID-19. This reflects the increasing COVID-19 testing capacity in Benin. In fact, the Benin Government, with support of the World Bank, World Health Organization, and several other local and international partners, has modernized and equipped the haemorrhagic viral fevers laboratory of Cotonou (initially the only diagnostic laboratory for COVID-19). Furthermore, 12 other laboratories capable of COVID-19 testing have been established, with state-of-the-art equipment, reagents, extraction kits, sampling kits and personal protective equipment, in order to increase its testing capacity. A toll-free telephone number was created to inform and direct people to sorting and screening centers that have gradually been opened in all 77 communes of the country. Screening sites have been set up at airports and borders, and mobile screening teams now visit administrative institutions and businesses. In addition, a screening network has been set up to detect, alert and track suspected COVID-19 cases throughout the country [11,12]. Our study has several limitations. The number of participants was quite small and respondents cannot be considered as representative of the general population. The high adherence to the preventive measures may be because the majority of respondents had a university level of education. It is important to conduct additional studies among the less educated who may be less likely to adhere to preventive measures.

## Conclusion

So far the COVID-19 pandemic does not seem to have caused a very high COVID-19 morbidity and mortality in Benin. Whether this is related to the implementation of the COVID-19 preventive measurements by the government and good adherence of the population to these measures needs to be investigated. Larger population surveys of all parts of society and qualitative research is needed to assess the preventive behavior of the population and the consequences of the pandemic on people´s well-being. People who obtained COVID-19 information from official governmental sources were more adherent to the COVID-19 preventive measures. Therefore, authorities must intensify their communication strategies to inform the population about the fight against this pandemic to avoid an increase in the number of COVID-19 cases or a second epidemic wave.

### What is known about this topic

- The implementation of COVID-19 mitigation measures may control a COVID-19 outbreak;
- To mitigate COVID-19 outbreak impacts, the Benin government opted for a sanitary cordon around the affected regions with free movement of people while respecting hand hygiene and barrier measures;
- So far the COVID-19 pandemic does not seem to have caused a very high COVID-19 morbidity and mortality in Benin.

### What this study adds

- Adherence to COVID-19 preventive measures in Benin, seemed satisfactory;
- Increasing age and obtaining COVID-19 information from official sources were significantly associated with higher adherence scores in a multivariable model;
- A wide dissemination of adequate information about COVID-19 would increase adherence, and targeted efforts should be directed towards increasing the adherence to preventive measures among the younger age groups.
